# Cystic Fibrosis Mortality in Childhood. Data from European Cystic Fibrosis Society Patient Registry

**DOI:** 10.3390/ijerph15092020

**Published:** 2018-09-15

**Authors:** Anna Zolin, Anna Bossi, Natalia Cirilli, Nataliya Kashirskaya, Rita Padoan

**Affiliations:** 1 Department of Clinical Sciences and Community Health, University of Milan, Milano 20133, Italy; anna.bossi@unimi.it; 2 Cystic Fibrosis Centre, Mother-Child Department, United Hospitals, Via Conca, 71, Torrette di Ancona I-60126, Italy; natalia.cirilli@ospedaliriuniti.marche.it; 3 Laboratory of Genetic Epidemiology, Federal State Scientific Budgetary Institution «Research Centre for Medical Genetics», Moscow 115522, Russia; kashirskayanj@mail.ru; 4 Cystic Fibrosis Unit, Pediatric Department, ASST Spedali Civili Brescia, piazzale Spedali Civili, Brescia 25123, Italy; ritaf54@gmail.com

**Keywords:** mortality, childhood, lung disease, cystic fibrosis, registry

## Abstract

Data collected in the European Cystic Fibrosis Society Patient Registry (ECFSPR) database were used to investigate whether risk factors for death in childhood and adolescents CF patients have different impact in countries of different income. In this way, it is possible to recognize where interventions could improve the quality of care and survival in these patients. We matched deceased and alive patients by age, country, year of follow-up. Multivariable logistic models were developed. In the years of this study, the ECFSPR collected information on 24,416 patients younger than 18 years: 7830 patients were from countries with low/middle income and 16,586 from countries with high income; among these the dead are 102 and 107 (*p* < 0.001), respectively. The use of oxygen, forced expiratory volume in one second (FEV_1_) below 40% and BMI standard deviation score (SDS) below −2 represent risk factors for death. However, some patients from countries with high income remain alive even if their values of FEV_1_% and BMI-SDS were low, and some deceased patients from countries with high income had high values of FEV_1_% (>60%). Evaluation of mortality in pediatric age may reflect the availability of resources for CF diagnosis and treatment in some countries.

## 1. Introduction

Recent reports from Australian, U.S. and European cystic fibrosis (CF) registries [1,2,3] show that adult patients represent more than 50% of the CF population, and the mean age of CF population is slowly but steadily increasing. At the same time, 2014 data from registries report a proportion of death in paediatric age ranging from 10% to 20% [Australia = 10.5% (two out of 19 patients); U.S. = 11.9% (55 out of 461); EU = 17.7% (68 out of 385)].

Since pulmonary insufficiency is the main cause of death in CF, the potential risk factors for pulmonary disease, which are to some extent preventable or treatable, could also be considered potential risk factors for death.

Kerem et al. [4] investigated the associations between forced expiratory volume in one second (FEV_1_) and clinical outcomes in the European Cystic Fibrosis Society Patient Registry (ECFSPR) database. BMI, infection by *Pseudomonas aeruginosa*, pancreatic status and CF-related diabetes (CFRD) showed a statistically significant and clinically important effect on FEV_1_. Recently, McColley et al. [5] identified respiratory signs and symptoms at age 3–5 years as risk factors for childhood death.

The paper of Mehta et al. [6] stated that mortality in childhood may be considered as a surrogate marker for the ability of a country’s health care system to provide early care to patients with severe chronic disease. The paper of McCormick et al. [7] highlighted the lack of studies on childhood mortality. The aim of this study is to investigate whether the risk factors for death reported in literature have different impact in countries of different income, so as to recognize the areas where interventions could improve the quality of care and survival of CF patients.

## 2. Patients and Methods

The ECFSPR annually collects data on CF patients from national registries and individual centres in Europe and neighbouring countries. The ECFSPR structure, the methods of collection, and management of the data are described elsewhere [8,9]. The self-reported coverage varies between the countries and years of follow-up [10].

We considered patients <18 years registered in the ECFSPR from 2008 to 2013 (the most recently available year of follow-up at the time of this analysis). We selected patients who died, then we matched (1:1) deceased and alive patients by age (range of two months, age at death for deceased patients, age at follow-up for alive patients), country and year of follow-up. We considered these variables for the matching to account for temporal and spatial differences in the diagnosis and treatment of disease and data collection. The matched alive patient was randomly selected among those matching the deceased patient. 

Countries were classified into three groups based on tertiles of gross national income per capita, obtained from World Bank tables [11]. Low and middle income countries were put together in one group having a number of deaths under 18 years similar to that of high income countries.

The ECFSPR collects information on demographics, diagnosis, genetics, infections, therapies, complications, lung function, growth and transplants. The definition of the variables recorded in the ECFSPR are given in the ECFSPR webpage [12]. The slight differences between countries in the definition of some variables do not affect the relationship between risk factors and mortality.

FEV_1_ values were expressed as percent of predicted (FEV_1_%) according to Wang et al. [13] for male and female children older than 6 years, and Hankinson et al. [14] for males and females aged ≥ 16 years. BMI standard deviation scores (SDS) of patients aged ≥ 2 years were computed on the basis of the Centres for Disease Control and Prevention references [15], and recoded as BMI-SDS < −2 and BMI-SDS ≥ −2. Due to the wide differences in the liver involvement in CF, patients were classified as “Cirrhosis with or without hypertension/hypersplenism, or hypertension unknown” or “Liver disease without cirrhosis”.

All patients gave their informed consent for inclusion before they participated to the ECFSPR. The study was conducted in accordance with the Declaration of Helsinki, and the data request was approved by the Scientific and Steering Committees of the ECFSPR, and anonymous patient data were provided for analysis (approval: 19/02/2016). The use of the provided data was according to Danish law.

### Statistical Analysis

To investigate which risk factors had an effect on the probability of death, a two steps strategy was adopted. In the first step, all factors, among those recorded in the ECFSPR, were grouped in five classes:

(1) Diagnosis: Genotype, Age at diagnosis, Neonatal screening, Meconium ileus
(2) Microbiology: Chronic Pseudomonas aeruginosa, Chronic Staphylococcus aureus, Chronic Burkholderia cepacia, Nontuberculous mycobacteria, Stenotrophomonas maltophilia
(3) Therapies: Inhaled hypertonic NaCl, Inhaled antibiotic, Inhaled bronchodilators, Use of oxygen, Use of rhDNase, Use of macrolide, Use of ursodeoxycholic acid
(4) Complications: Allergic bronchopulmonary Aspergillosis (ABPA), CFRD, Pneumothorax requiring chest drain, Liver disease, Hemoptysis, Occurrence of malignancy
(5) Growth and lung function: BMI-SDS, FEV_1_%

A multivariable logistic model adjusted by gender was used in each class (model 1–5) to select the risk factors significantly associated to mortality. In the second step the selected factors were entered into a multivariable logistic model (model 6.1). Since BMI can be computed only for patients older than 2 years and FEV_1_% for patients older than 6 years, we entered these variables as the last (model 6.2) to not narrow the age of the patients during the variable selection process. The same analysis was performed on the two subgroups of countries, low/middle income and high income, in order to investigate if the risk factors have different impact in different countries.

## 3. Results

For years 2008–2013, the ECFSPR collected information on 44,104 CF patients from 27 countries. In these years, the total number of deaths was 1754 (3.97%). For the purposes of the study we considered 24,625 patients (24,416 alive and 209 deceased) younger than 18 years with ascertained status of life (patients not seen during the years of follow-up or lost to follow-up were excluded). 

Out of the 209 deceased patients, only 186 deceased patients had one matching alive patient. 

The most frequent cause of death was respiratory disease (57.0%), followed by unknown cause (16.7%, this category can include rare complications: only a limited number of cause of death is collected by the ECFSPR), transplantation (10.2%), non-CF related causes (10.2%), liver or gastro-intestinal disease (4.3%), trauma (1.6%). 11.83% of patients died before their second birthday and 67.75% died between 10 and 17 years. 65.59% of the deceased patients were female: both for males and females more than 60% of the patients died between 10 and 17 years.

As expected, cases with severe disease were more frequent in deceased patients (Table 1).

**Figure 1:**
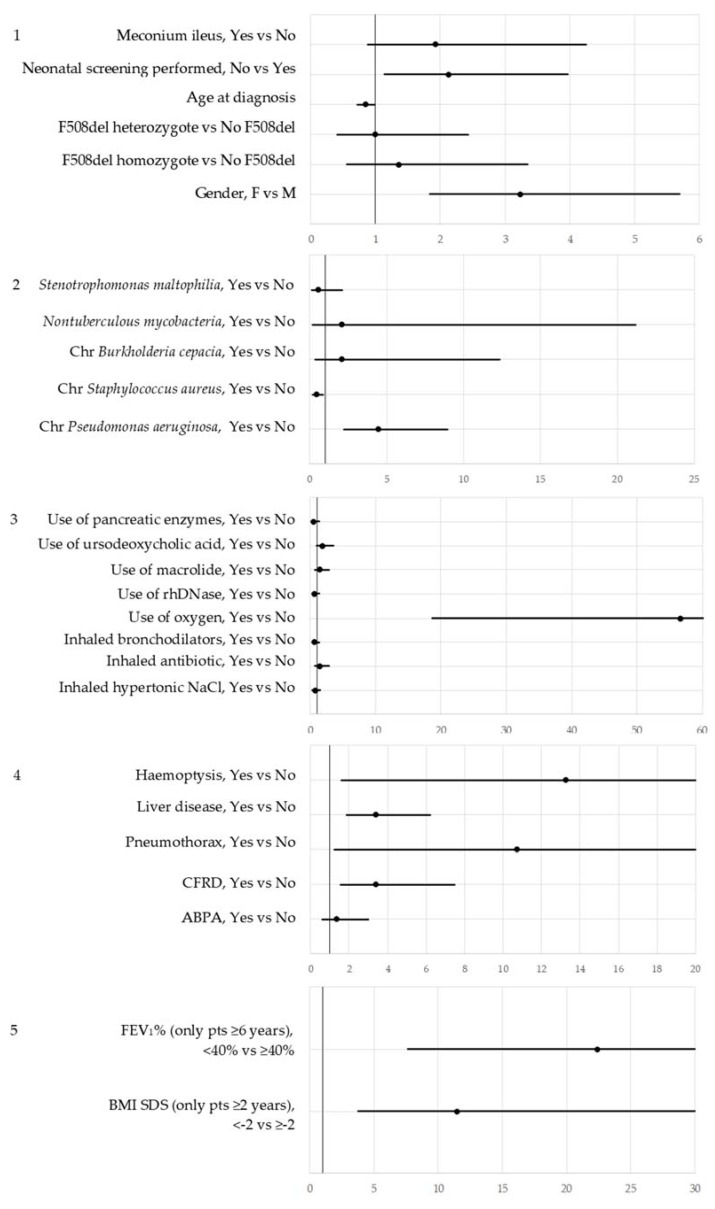
Results of the first step strategy: they are expressed as OR and 95% confidence interval (CI). All factors, among those recorded in the ECFSPR, were grouped in five classes: diagnosis (**1**), microbiology (**2**), therapies (**3**), complications (**4**), growth and lung function (**5**), Chr: chronic.

Gender, age at diagnosis, neonatal screening, chronic *Pseudomonas aeruginosa*, chronic *Staphylococcus aureus*, oxygen supplementation, CFRD, pneumothorax requiring chest drain, liver disease, haemoptysis, BMI and FEV_1_ resulted significantly associated to mortality (Figure 1).

All these variables were considered in the second step of the analysis, except haemoptysis and pneumothorax requiring chest drain (only 1 alive patient with one of these conditions). Among these variables (except BMI-SDS and FEV_1_%, available only for patients over 2 and 6 years, respectively), when simultaneously included into a logistic model (model 6.1), only gender and oxygen supplementation resulted significantly associated to mortality. BMI-SDS and FEV_1_%, added to the model (model 6.2), appeared significantly associated to mortality, with a reduced effect of gender and use of oxygen (Table 2).

The interaction between BMI and FEV_1_ was not significant. In the years of this study, the ECFSPR collected information on 12,867 patients from 17 countries with low/middle income and 31,237 patients from 10 countries with high income. Seven thousand eight hundred thirty patients younger than 18 years were from countries with low/middle income and among these 102 died (1.30%); 16,586 patients younger than 18 years were from countries with high income and among these 107 died (0.64%). The difference was statistically significant (*p* < 0.001).

Lithuania reported no data on children or adolescent patients and for Latvia and Republic of Moldova deceased patients did not have a living matching patient. 5 countries do not report deceased patients. Therefore, the 372 patients included in the analysis were from 19 countries (Table 3).

To investigate if the risk factors for death differed across European countries, we performed the same analysis considering the two subgroups of countries, high income and low/middle income. Since the number of patients considered for these models is small, the 95% CI is too large, therefore we decided to report only the low limit of it: in this way we report an estimate of the minimum risk. For the group of countries with high income, use of oxygen, FEV_1_% and BMI-SDS were associated with the probability of death. The odds of dying was 4.7 (low limit of the 95% CI of the OR) times higher in patients that use oxygen, the odds of dying was 1.2 times higher in patients with FEV_1_% below 40%; patients with a BMI-SDS below −2 had an odds of dying 2.9 times higher than that of patients with a BMI-SDS above −2 SDS. 

For the group of countries with low/middle income, oxygen supplementation was not significant and no longer considered into the final model. The odds of dying was 6.7 (low limit of the 95% CI of the OR) times higher in patients with FEV_1_% below 40% than in patients with FEV_1_% above or equal to 40%. Patients with a BMI-SDS below −2 had an odds of dying that was 1.3 (low limit of the 95% CI) times higher than patients with a BMI-SDS above −2 SDS.

**Figure 2:**
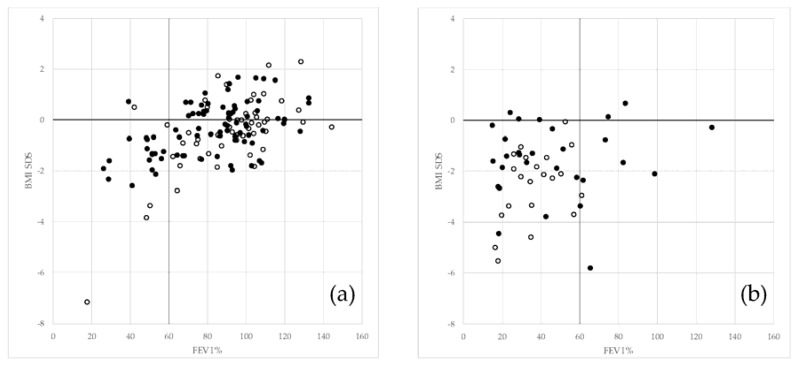
(**a**): alive patients from low/middle income countries (empty bullet) and from high income countries (circle bullet) by BMI and lung function. (**b**): deceased patients from low/middle income countries (empty bullet) and from high income countries (circle bullet) by BMI and lung function.

Figure 2 shows the distribution of living (2a) and deceased (2b) patients by lung function and nutrition in low/middle and high income countries. Some patients from countries with high income remain alive even if their values of FEV_1_% and BMI-SDS were low (<60% and <0, respectively). Some deceased patients from countries with high income had high values of FEV_1_% (>60%). 

## 4. Discussion

Our study investigated risk factors for death in patients younger than 18 years using ECFSPR data. The childhood mortality may be considered as a surrogate marker for the ability of a country’s health care system [6]. For this reason, we investigated whether the risk factors for death have different impact in countries of different income for recognizing where interventions could improve the quality of care and survival of CF patients.

In the ECFSPR, the percentage of death in paediatric age (11.9%) is similar to those reported in the Australian registry [1] and in the CF Foundation (CFF) Patient Registry [2]. McColley et al. [5] using clinical data from the Epidemiologic Study of CF [16] showed that also digital clubbing or chest crackles between 3 and 5 years of age, both signs of early severe pulmonary involvement, associated with worse lung disease, are risk factors for death.

According to literature, among the risk factors for early death considered in the first step of our analysis, those resulted significantly associated with higher probability of death are: female gender, high age at diagnosis, unavailability of newborn screening (NBS), chronic infection by *Pseudomonas aeruginosa* or by *Staphylococcus aureus*, use of oxygen, CFRD, pneumothorax requiring chest drainage, liver disease, haemoptysis, BMI-SDS below−2 and FEV_1_% below 40%. As expected, except for gender, age at diagnosis and NBS, the major risk factors identified in this study, as well as in literature, are CF complications. 

An OR significantly below 1 for the age at diagnosis and significantly above 1 for the unavailability of NBS means that in the absence of a CF NBS program, the early appearance of symptoms should be considered a negative prognostic factor, and probably patients diagnosed later have a milder disease. The diagnosis due to a positive NBS seems to be a protective factor from early death [17] even for severe CF.

When the variables found to be significantly associated with mortality in the first step of our analysis were simultaneously included in a multivariable model, respiratory failure (need for oxygen and FEV_1_% < 40%) and malnutrition (BMI-SDS < −2) emerged as significantly associated with death. Gender appeared associated with mortality: we observe an OR of 4.118, suggesting that female gender could be a risk factor. The reason for the gender gap in CF is still not clear [18] although several hypotheses have been formulated [19,20,21,22].

Chronic *Pseudomonas aeruginosa* and *Staphylococcus aureus* respiratory infections, presence of diabetes or liver disease are no longer significant: indeed, all these CF features are reported to negatively influence both nutritional status and respiratory function [23]. However, recommendations for early detection and treatment of the severe form of lung disease in children, together with timing for referring to lung transplant, are still lacking [24,25].

It is noticeable that the prevalence of CFRD among CF deceased patients was higher than among CF living patients (24.2% vs. 7%). Conversely, as prevalence of CFRD is less than 5% in the whole CF paediatric population reported in the ECFSPR, a wider dissemination of paediatric diabetes screening and therapy guidelines in CF subjects is desirable, in order to achieve early treatment [26].

We can consider that risk factors not included in the final model have effect on the worsening of both the pulmonary function (need for oxygen and FEV_1_% < 40%) and the nutritional status, therefore these two factors are sufficient to identify patients at risk.

Kerem et al. [4] showed BMI has the greatest impact on lung disease. Patients with poor BMI experienced a six-fold increased odds of severe lung disease. Our results confirmed that almost (87%) all the deceased patients younger than 18 years had a BMI-SDS less than 0; so the importance of good nutritional status in childhood should be emphasized.

Guidelines concerning diagnosis and treatment in NBS positive CF infants [27], and guidelines about the management of nutritional problems, diagnosis and treatment of diabetes in the paediatric age [28,29] are available. A paper [30] gives evidence-based consensus recommendations for the care of CF preschool children on routine surveillance for pulmonary disease, therapeutics and nutritional and gastrointestinal care. Moreover, a good nutritional status in early childhood is associated with improved survival [31].

The same analysis, performed by country grouping, came at the same conclusions as the analysis considering all the countries together: only the use of oxygen, FEV_1_% < 40% and BMI-SDS < −2 were found to be significantly associated with death. However, the percentage of deceased patients younger than 18 years (over the total population < 18 years), is higher for countries with low/middle income (1.3%) than for high income countries (0.64%). Evaluation of mortality in paediatric age may reflect the availability of resources for CF diagnosis and treatment. However to check this hypothesis further ad hoc studies are needed. Furthermore, we observed that some deceased patients from countries with high income had higher values of FEV_1_% and of BMI. On the contrary, some patients were still alive even if their values of FEV_1_% and BMI were very low. It should be noted that the FEV_1_% value does not always reflect the severity of the lung disease, as shown by studies on thoracic imaging using CT scan [32]. Our results can also be explained by the complexity of both CF disease and its management. As discussed by Oates et al. [33] several non-genetic factors including health care can influence CF disease progression and outcomes.

The study has some limitations: Macedonia, Moldova and Romania (low income), and Latvia, Slovakia and Slovenia (middle income) do not report patients who died in childhood/adolescenthood. Furthermore, as regards Lithuania (low income), ECFSPR does not have any information on CF patients in the paediatric age. ECFSPR data might not properly describe the mortality of CF populations younger than 18 years for countries with low/middle income: these can be countries where CF diagnosis is difficult and where patients can die before receiving the correct diagnosis. 

## 5. Conclusions

Based on our results we might no more define CF as a lethal disease in childhood, at least in patients followed by specialized care centres. Our results highlight the need for quality of care improvements: health policies interventions that promote early diagnosis of CF patients through NBS programmes [34,35] should be encouraged and the performance of existing NBS programmes carefully monitored [36]. Early diagnosis can preserve lungs from damage in childhood, contributes to a better nutritional status with improved growth [31] and reduces the burden of care for families and may improve survival [17]. 

The identification of paediatric patients at risk of death through FEV_1_ < 40% and use of oxygen might be too tardive in order to perform useful treatment actions to change the prognosis. Therefore, CF centres should implement diagnostic tools to identify patients at risk earlier, using lung CT scan and bronchoalveolar lavage; lung clearance index [37] to identify those patients who need more intensive nutritional and respiratory care. As a consequence, guidelines for the diagnosis and treatment of patients with early onset of severe respiratory disease, aggressive nutritional support for CF patients and improvement in early diagnosis of CFRD are desirable in the paediatric settings.

Due to the importance of this topic, these results should be shared and deeply discussed with health care authorities in each country in order to orient health policies in terms of prevention. The results obtained have some limitations: it will be interesting to perform a new analysis on ECFSPR mortality data in paediatric age. An ad hoc study can help to identify those countries that can benefit from interventions to improve the quality of care and survival of CF patients.

## Figures and Tables

**Table 1:** Main demographic and clinical characteristics of the 372 considered patients. The number of patients and the percentage (in brackets) were reported unless otherwise stated.

			Deceased (N = 186)	Alive (N = 186)
	Gender	Male Female	64 (34.41) 122 (65.59)	109 (58.60) 77 (41.40)
Diagnosis	Genotype	F508del homozygote F508del heterozygote No F508del Unknown	87 (46.77) 66 (35.48) 25 (13.44) 8 (4.30)	83 (44.62) 73 (39.25) 25 (13.44) 5 (2.69)
	Age at diagnosis (years), median (N)		0.30 (175)	0.31 (174)
	Age at diagnosis	<1 year 1–17 years Unknown	132 (70.97) 43 (23.12) 11 (5.91)	114 (61.29) 60 (32.26) 12 (6.45)
	Neonatal screening	Not performed Performed Unknown	87 (46.77) 36 (19.35) 63 (33.87)	79 (42.47) 53 (28.49) 54 (29.03)
	Meconium ileus	No Yes Unknown	130 (69.89) 37 (19.89) 19 (10.22)	145 (77.96) 23 (12.37) 18 (9.68)
Transplant	Liver transplant	No Yes Unknown	178 (95.70) 1 (0.54) 7 (3.76)	180 (96.77) 0 (0.00) 6 (3.23)
	Lung transplant	No Yes Unknown	152 (81.72) 30 (16.13) 4 (2.15)	182 (97.85) 0 (0.00) 4 (2.15)
Microbiology	Chronic *Pseudomonas aeruginosa*	No Yes Unknown	73 (39.25) 77 (41.40) 36 (19.35)	132 (70.97) 38 (20.43) 16 (8.60)
	Chronic *Staphylococcus aureus*	No Yes Unknown	66 (35.48) 31 (16.67) 89 (47.85)	71 (38.17) 45 (24.19) 70 (37.63)
	Chronic *Burkholderia cepacia*	No Yes Unknown	142 (76.34) 12 (6.45) 32 (17.20)	167 (89.78) 2 (1.08) 17 (9.14)
	*Nontuberculous mycobacteria*	No Yes Unknown	139 (74.73) 4 (2.15) 43 (23.12)	151 (81.18) 3 (1.61) 32 (17.20)
	*Stenotrophomonas maltophilia*	No Yes Unknown	141 (75.81) 9 (4.84) 36 (19.35)	146 (78.49) 18 (9.68) 22 (11.83)
Therapy	Inhaled hypertonic NaCl	No Yes Unknown	115 (61.83) 37 (19.89) 34 (18.28)	129 (69.35) 42 (22.58) 15 (8.06)
	Inhaled antibiotic	No Yes Unknown	72 (38.71) 82 (44.09) 32 (17.20)	118 (63.44) 59 (31.72) 9 (4.84)
	Inhaled bronchodilators	No Yes Unknown	56 (30.11) 95 (51.08) 35 (18.82)	78 (41.94) 93 (50.00) 15 (8.06)
	Use of oxygen	No Yes Unknown	70 (37.63) 83 (44.62) 33 (17.74)	169 (90.86) 4 (2.15) 13 (6.99)
	Use of rhDNase	No Yes Unknown	58 (31.18) 103 (55.38) 25 (13.44)	74 (39.78) 107 (57.53) 5 (2.69)
	Use of macrolide	No Yes Unknown	75 (40.32) 74 (39.78) 37 (19.89)	120 (64.52) 51 (27.42) 15 (8.06)
	Use of ursodeoxycholic acid	No Yes Unknown	68 (36.56) 87 (46.77) 31 (16.67)	99 (53.23) 82 (44.09) 5 (2.69)
	Use of pancreatic enzymes	No Yes Unknown	13 (6.99) 146 (78.49) 27 (14.52)	22 (11.83) 160 (86.02) 4 (2.15)
Complication	ABPA	No Yes Unknown	137 (73.66) 25 (13.44) 24 (12.90)	160 (86.02) 16 (8.60) 10 (5.38)
	CFRD	No Yes Unknown	120 (64.52) 45 (24.19) 21 (11.29)	168 (90.32) 13 (6.99) 5 (2.69)
	Pneumothorax requiring chest drain	No Yes Unknown	154 (82.80) 11 (5.91) 21 (11.29)	179 (96.24) 1 (0.54) 6 (3.23)
	Liver disease	No Yes Unknown	95 (51.08) 64 (34.41) 27 (14.52)	145 (77.96) 25 (13.44) 16 (8.60)
	Haemoptysis	No Yes Unknown	145 (77.96) 17 (9.14) 24 (12.90)	176 (94.62) 1 (0.54) 9 (4.84)
	Occurrence of malignancy	No Yes Unknown	158 (84.95) 1 (0.54) 27 (14.52)	173 (93.01) 0 (0.00) 13 (6.99)
Growth and lung function	BMI-SDS (only patients 2 years old or more)	<−2 ≥−2 Unknown	45 (27.44) 49 (29.88) 70 (42.68)	10 (6.13) 145 (88.96) 8 (4.91)
	FEV_1_% *	<40 ≥40 Unknown	28 (23.33) 22 (18.33) 70 (58.33)	6 (4.03) 128 (85.91) 15 (10.07)

* excluding patients under 6 years of age and those who received a lung transplant.

**Table 2:** Effect of selected factors on mortality in the set of patients under 18, and in the subset of patients 6 to 18 years old.

	OR	95% CI	
Model 6.1: 136 observations used			
Gender: F vs. M	5.626	1.878	16.853
Neonatal screening: Not performed vs. Performed	0.755	0.235	2.421
Use of oxygen: Yes vs. No	48.608	8.173	289.070
CFRD: Yes vs. No	3.646	0.832	15.991
Liver disease: Yes vs. No	1.935	0.673	5.568
Chronic *Pseudomonas aeruginosa*: Yes vs. No	2.512	0.852	7.410
Chronic *Staphylococcus aureus*: Yes vs. No	0.483	0.172	1.353
Age at diagnosis	0.835	0.604	1.153
Model 6.2: 172 observations used			
Gender: F vs. M	4.118	1.211	14.005
Use of oxygen: Yes vs. No	20.787	4.336	99.668
BMI-SDS (patients aged 2 or more): <−2 vs. ≥−2	7.975	2.403	26.461
FEV_1_% *, <40 vs. ≥40	6.999	1.997	24.524

* excluding patients under 6 years of age and those who received a lung transplant.

**Table 3:** ECFSPR countries by group of income.

		Number of Ptients		
Income	Countries	total	total < 18 years	deaths < 18 years
Low/middle	Hungary, Lithuania, Republic of Macedonia *, Republic of Moldova, Romania *, Russian Federation, Serbia, Ukraine, Czech Republic, Greece, Israel, Italy, Latvia, Portugal, Slovakia *, Slovenia * and Spain	12,867	7830	102
High	Austria, Belgium, Denmark, France, Germany, Ireland, The Netherlands, Sweden, Switzerland * and United Kingdom	31,237	16,586	107

* no deceased patients from this country.
